# A modified Park's stitch to correct aortic insufficiency for bioprosthetic valve at time of left ventricular assist device implant: a case report

**DOI:** 10.1186/s13019-016-0555-3

**Published:** 2016-11-30

**Authors:** Toshinobu Kazui, Nicole Sydow, Mark Friedman, Samuel Kim, Scott Lick, Zain Khalpey

**Affiliations:** 1 Division of Cardiothoracic Surgery, The University of Arizona/ Banner University Medical Center Tucson, 1501 N Campbell Ave., Rm 4302A, P.O. Box 245071, Tucson, AZ 85724-5071 USA; 2 Division of Cardiology, The University of Arizona/ Banner University Medical Center Tucson, Tucson, AZ USA

**Keywords:** Aortic insufficiency, Aortic valve repair, Park’s stitch, Bioprosthetic valve, Left ventricular assist device

## Abstract

**Background:**

Aortic valve insufficiency (AI) at the time of left ventricular assist device (LVAD) insertion needs to be corrected, however there is little known about how to manage bioprosthetic valvular AI.

**Case presentation:**

A 55-year-old female with dilated cardiomyopathy who previously had a bioprosthetic aortic valve replacement needed a LVAD as a bridge to transplant. Her left ventricular ejection fraction was 10% and had mild to moderate transvalvular AI. She underwent a HeartWare HVAD insertion along with aortic valvular coaptation stitch repair (Park's stitch) to the bioprosthetic valve.

**Conclusion:**

Her AI improved to trivial with minimal ejection through the bioprosthetic valve. She was transplanted 6 months following the surgery. A Park's stitch to the bioprosthetic aortic valve with more than mild AI might be a good option for bridge to transplant patient.

## Background

Continuous flow left ventricular assist devices (LVADs) are known to cause progressive aortic valve commissural fusion as well as progressive aortic insufficiency with greater pump speeds and larger aortic root dimensions [1, 2]. Currently, consensus is to intervene on the aortic valve at the time of LVAD implant if the patient has more than mild aortic insufficiency (AI) (Class I, Level of Evidence C) [3]. The aortic valve can either be repaired or replaced with a bioprosthetic valve. The central coaptation repair stitch (Park’s stitch) has been shown to effectively and durably reduce the AI as well as improve survival, while maintaining aortic valve opening to allow ejection, without the additional cardiopulmonary bypass time that would be required for bioprosthetic valve replacement [4, 5]. This technique has typically been applied to native aortic valves in patients who present with significant AI at the time of LVAD implantation, but there is little known about the application of this technique to patients who present with bioprosthetic transvalvular aortic valve insufficiency at time of LVAD implant. This report demonstrates first clinical application of a Park’s stitch to a patient with a previous bioprosthetic aortic valve replacement (AVR) developing transvalvular AI.

## Case presentation

A 55 year-old female with non-ischemic cardiomyopathy who initially presented with multiple syncopal episodes and increasing shortness of breath on exertion for the preceding 2 months. Her left ventricular ejection fraction (LVEF) was 20% at that time, with severe aortic stenosis and mild AI. Her aortic valve area was 1.0 cm^2^, with a peak gradient 16.9 mmHg and mean gradient 9.2 mmHg gradient. She underwent AVR with a 21 mm Mitroflow (Sorin, Milan, Italy) bioprosthetic valve with significant improvement in her symptoms (aortic valve area 1.8cm^2^, peak gradient 18.2 mmHg and mean gradient 9.0 mmHg).

**Fig. 1:**
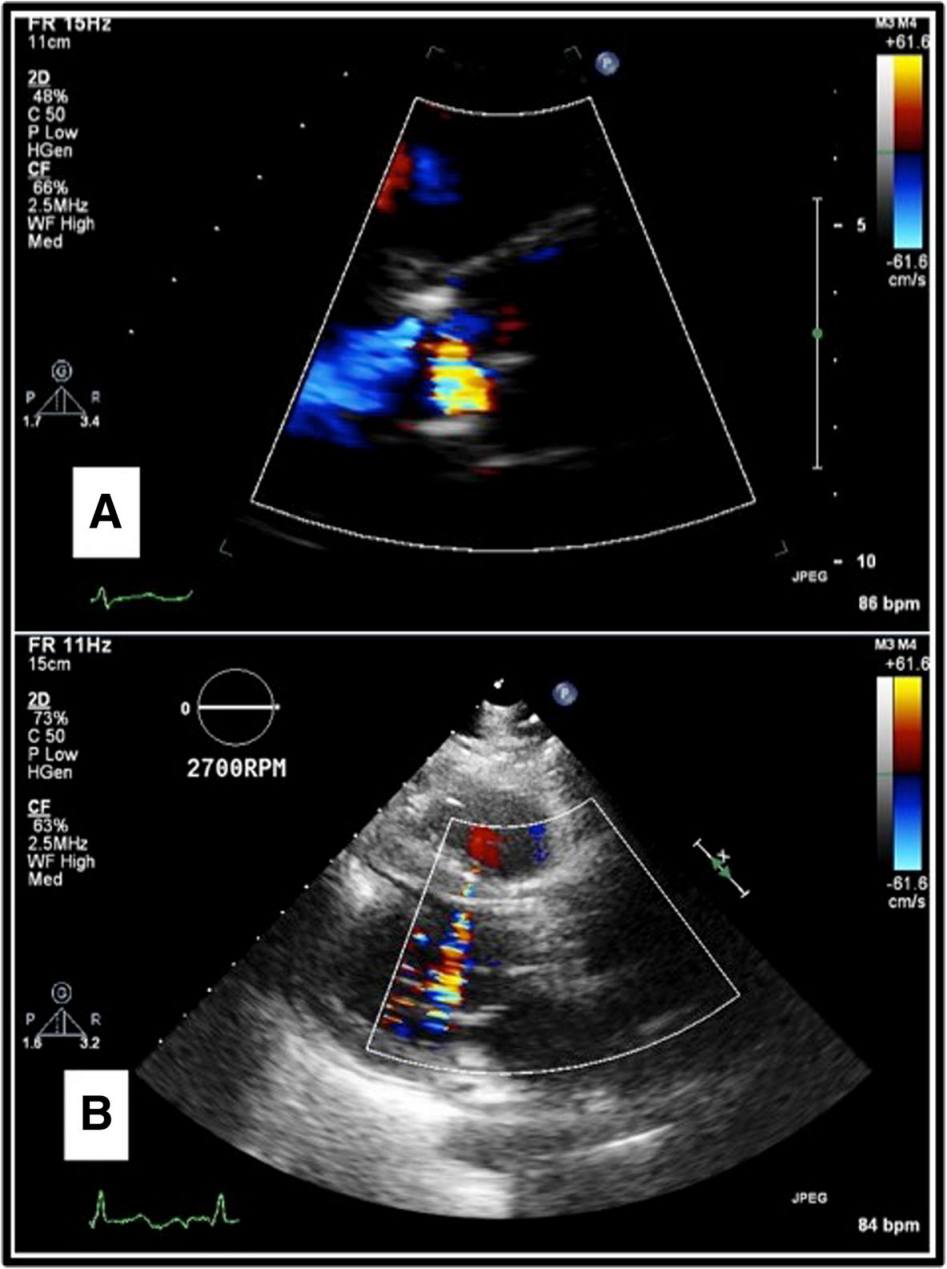
Pre and post op aortic valve insufficiency. There was intra-valvular aortic insufficiency in pre op aortic valve (**a**), no aortic insufficiency was noted after aortic valve coaptation stitch (**b**)

**Fig. 2:**
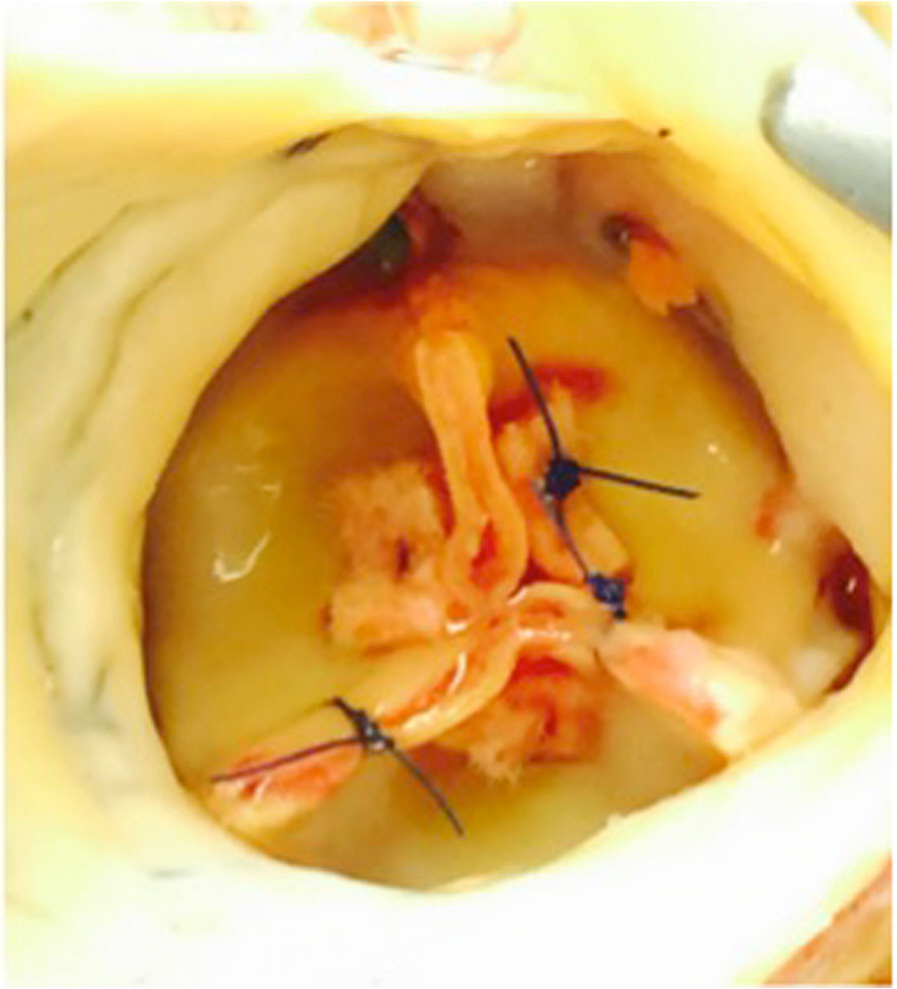
The prosthetic aortic valve at the time of heart transplant. Aortic valve leaflets were fused. No thrombus formation was confirmed *above* and *below* the valve

Over the next 2 months, the patient had worsening heart failure with a decline in her LVEF to 15%. Her medical regimen was optimized with some improvement in her symptoms initially, however the improvement was short lived. Subsequent echocardiograms showed 15% LVEF, with new mild to moderate intra-valvular AI and mild perivalvular leak. Seven months after her AVR the patient was severely short of breath with declining ventricular function and mild to moderate AI. Her right ventricular function was severely depressed. The decision was made to insert HeartWare HVAD (Framingham, MA) with prosthetic aortic valve repair, Park’s stitch. Her INTERMACS profile was 3. Her pre operative platelet count, prothrombin time, and INR were 138 (1000/μl), 14.5 s, and 1.1. At the time of her LVAD insertion, the repair stitch was able to minimize her ischemic time to help preserve her RV. A pledgeted 4-0 Prolene (Ethicon, Cincinnati, OH) was placed to center of the each aortic valve leaflet. We could not appreciate perivalvular leak intraoperatively, however pledgeted mattressed 2-0 ethibond sutures were passed through from subvalvular leaflets to aortic wall and tied over pledgets. No obvious leaflet prolapse or perforation was noted (Fig. 1). The cardiopulmonary bypass time was 167 min and myocardial ischemic time was 52 min. Post-operatively, the patient had no AI on transesophageal echocardiogram as well as limited ejection through the aortic valve. The anticoagulation management was aspirin 325 mg/day and continuous heparin infusion based on thromboelastogram until Coumadin gets therapeutic, INR 2-3. She recovered well and was discharged home at postoperative day 27. At 4 months post HVAD insertion, the patient was feeling well with only trace AI on transthoracic echocardiogram. She developed lacunar infarction within the ventromedial nucleus of the right thalamus on 3 months after LVAD insertion. She was heart transplanted at 6 months following her LVAD insertion. Her aortic valve was fused and closed. No thrombus formation underneath the aortic valve was noted (Fig. 2).

## Conclusion

Little has been known about management of recurrent AI after bioprosthetic AVR at the time of LVAD placement. A central coaptation stitch to these patients potentially is a good option for a bridge to transplant.

Short-term follow up in this patient shows promising results with only trace AI on echocardiogram. Although this technique has been shown to be a durable solution for patients with significant native aortic valve insufficiency [4], the long-term results of this technique especially in bioprosthetic valves remains to be seen to determine the durability of these results in the face of continued bioprosthetic valve degeneration.

Although there have been numerous studies of AI in LVAD patients, we don't know the long-term prognosis of patients who develops acquired AI. Since AI seemed to have negative impact on survival [1, 6], either bioprosthetic AVR or aortic valve repair is recommended for these patients. Patients with AI with following a previous bioprosthetic AVR, no recommendation is currently available. Performing a re-replacement bioprosthetic AVR may be more durable to prevent leaflet fusion, however explanting a previous valve and re-implant new valve in patients having an LVAD placement with concomitant severe RV dysfunction raises concerns about further impairing postoperative RV function. A Park's stitch to a bioprosthetic AVR is relatively easy, timely, safe and durable in LVAD patients being bridged to transplant.

This procedure was able to be performed in a quick and effective manner with short aortic cross clamp time to minimize the risk of RV dysfunction after LVAD placement.

The limitation of this technique in the setting of previous bioprosthetic AVR is no long-term mortality and morbidity data reported, therefore the application of this technique to destination patients should be cautious because of aortic valve leaflet fusion and potential thrombus formation below the fused aortic valve. If patients with AI from degenerating bioprosthetic AVR is listed as bridge to transplant, a Park's stitch to the bioprosthetic valve leaflets might be a good option to consider.

In conclusion, we reported a first successful case with Park's stitch and LVAD placement to previously placed bioprosthetic AVR. Further studies are mandatory to prove longevity and survival of this procedure in this context.
